# Supplementary material for: Development of a Patient Decision Aid for Rectal Cancer Patients with Clinical Complete Response after Neo-Adjuvant Treatment
Source: Cancers (Basel). 2023 Jan 28;15(3):806. doi: 10.3390/cancers15030806 (PMC9913303; doi:10.3390/cancers15030806)
Supplement: Supplementary file 1 [file cancers-15-00806-s001.zip › cancers-2076680-supplementary.pdf]

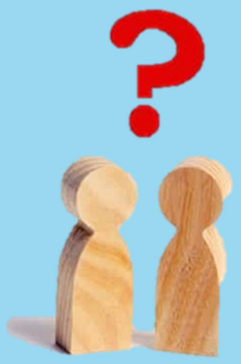

# “Surgery versus active surveillance”

NOT VALIDATED

Decision aid for patients with rectal cancer

INTRODUCTION 3
What is a decision aid? 3
Purpose of the decision aid 3
WHAT IS RECTAL CANCER? 4
TREATMENT OPTIONS 5
Option 1: Surgery 6
Option 2: Active surveillance 9
ADDITIONAL INFORMATION 10
SURVEY 11
PERSONAL CONSIDERATIONS 13
CONCLUSION 14
FIND OUT MORE? 15

NOT VALIDATED

NOT VALIDATED

This decision aid to choose between surgery and active surveillance only applies to you if:

- You have rectal cancer for which you have already received radiotherapy combined with chemotherapy;
- AND there is no visible tumor remaining after this treatment
- AND when your physician considers both options equivalent choices for you.

What is a decision aid?

- It helps you make a decision
- It informs you of the possible options
- It helps you compare possible options
- It helps you think about what is important to you

Purpose of the decision aid

In this decision aid you will find information about your disease (rectal cancer), treatment options and associated pros and cons. If you have enough information, you can weigh up the different treatment options based on the pros and cons. Finally, it helps you to engage in conversation with your doctor and actively ask questions. It may be useful to go through this decision aid together with your general practitioner and/or your relatives.

This decision aid is intended to supplement and not to replace the advice of your treating physicians.

## RECTAL CANCER

In 2020 there were 1.978 new cases of rectal cancer in Belgium. Of the patients 62% were men and 38% women.

The **rectum** is the last 15 cm of the colon just above the sphincter. It is an important part of the bowel because it is the reservoir in which stool is accumulated before you go to the toilet. The internal and external sphincter muscles coordinates bowel movements.

In rectal cancer, a malignant growth (= tumor) develops in the rectum. This may cause, among other things, an altered bowel movement pattern and loss of blood and mucus in the stool.

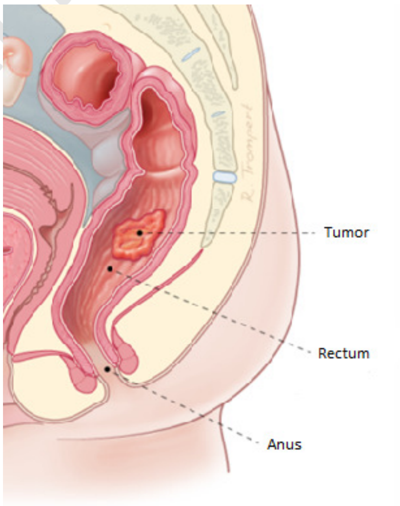


NOT VALIDATED

# TREATMENT OPTIONS

You have already been treated with radiotherapy and chemotherapy. Following this treatment, you underwent some more examinations (MRI scan, endoscopic examination, etc.) to evaluate the result of the treatment. These showed that the tumor has (almost) completely disappeared. As our scans and cameras are not microscopes, there is the possibility that a small number of cancer cells have remained. This uncertainty can be dealt with in two ways:

1) **Surgery**

2) Close monitoring of the rectum (= **active surveillance**) to detect a recurrence (return of the malignant tumor) early.

NOT VALIDATED

## OPTION 1: SURGERY

During **rectal resection**, the surgeon removes the site where the malignant tumor was located (affected piece) together with the surrounding fatty tissue. In most cases, this procedure is done laparoscopically (through small openings in the abdomen) or with the assistance of a robot. Part of the procedure may be done via the anus.

After surgery, you will be followed up with clinical examination, blood sampling and control scans according to the schedule below:

Follow-up schedule:

- The first 3 years: every 6 months
- 4th and 5th year: every 6 months/annually
- 6th year and thereafter: 1x/year
- Colonoscopy: 1 year after surgery and every 3 years thereafter

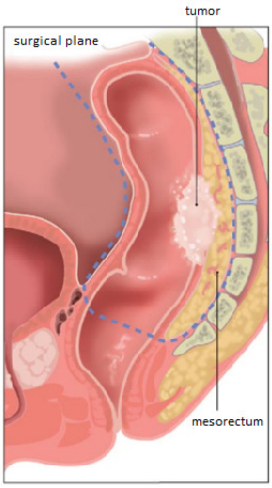


NOT VALIDATED

# OPTION 1: SURGERY

To ensure that the chance that the tumor recurs is minimal, part of the bowel above and below the tumor is also removed. After the 'affected' part of the rectum has been removed, the two healthy parts of the bowel are reconnected (= anastomosis). In this approach, the sphincter muscle is spared.

To allow the new connection to heal, the surgeon often places a temporary stoma on the small intestine. This is removed again at a later date.

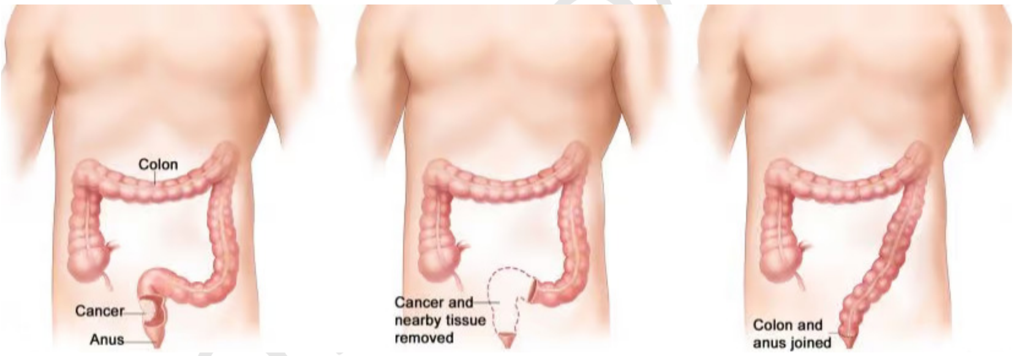


NOT UPDATED

# OPTION 1: SURGERY

If the tumor is located low in the rectum there is a possibility that not enough bowel remains to make a proper connection. In some cases the tumor may have grown into the sphincter. In this case, the sphincter is removed along with the tumor and a definitive stoma is created. This involves the divertion of the end of the colon through an opening in the abdominal wall to collect the stool in a pouch (=colostomy bag).

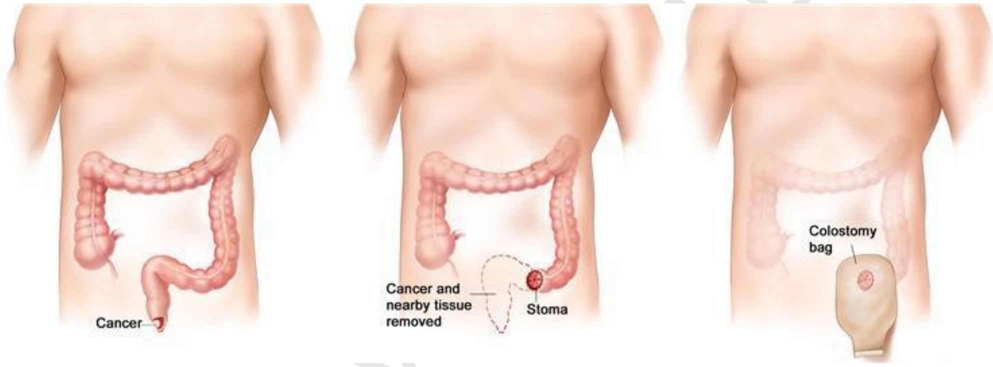


Each type of surgery may have consequences. These are explained in detail in 'overview'. The surgeon will discuss with you for which option you are eligible, for the operation with the temporary stoma or the one with the definitive stoma.

## OPTION 2: ACTIVE SURVEILLANCE

When the MRI and endoscopic examination no longer show any malignant tumor, there is a good chance that, due to pre-treatment, there are no cancer cells present anymore. Therefore, instead of surgery, active and close surveillance of the bowel and the rest of the body can also be an option.

If you opt for active surveillance, you will undergo no further chemotherapy, radiotherapy or surgery. You will be subjected to regular clinical examinations, control scans and endoscopic examinations to monitor the situation. Based on these examinations, we will check for signs of a recurrence of the rectal cancer. If all examinations are reassuring, further follow-up will be suggested.

Follow-up schedule:

- The first 3 years: every 3 to 4 months
- 4th en 5th year: every 6 months
- 6th year and thereafter: 1x/year

If there is a recurrence of the tumor in the rectum, surgery will still have to be performed. At that point, the doctor will discuss with you what type of surgery you need and whether it will lead to a temporary or permanent stoma.

NOT VALIDATED

# ADDITIONAL INFORMATION

**L**ow **A**nterior **R**esection **S**yndrome **(LARS)**

After completing treatment (neo-adjuvant treatment with or without surgery), you may experience that your bowel movement has changed. The problems with stool you may experience manifest themselves mainly in the form of incontinence, clustering, urgency and problems around frequency.

- Incontinence: reduced or loss of control over bowel movements and/or wind
- Frequency: increase in the number of times you have bowel movements per day
- Clustering: numerous bowel movements occurring within a short period
- Urgency: rushing to the toilet when you feel the urge to have bowel movements/sudden need to rush to the bathroom to empty the bowels

Symptoms may vary from patient to patient, in severity and duration.

NOT VALIDATED

| Surgery | Active surveillance |
|---|---|
| **Advantages** | |
| • Greater certainty of complete tumor elimination at intestinal level.<br>• Follow-up remains necessary to detect possible meta-stases. | • Spared the potential cons of surgery.<br>• Chances of cure are equally high with this strategy.<br>• Recurrence can be detected early by strict follow-up. |
| **Disadvantages** | |
| **WITHOUT stoma:**<br>Short term<br>• Wound infections (24%)<br>• Anastomosis leakage (5%)<br>• Bleeding at the level of the anastomosis (2%)<br>• Ileus (17%)<br>• Bowel movements may be delayed after surgery<br>Long term<br>• Stool problems (LARS)<br><br>**WITH stoma:**<br>Long term<br>• Skin problems and irritations<br>• Parastomal hernia<br>• Daily care of the stoma<br>• Prolapsed stoma<br>• Stoma leaks<br>• Adjusted feeding and drinking pattern<br>→ Above mentioned stoma problems occur in 3.4% of the patients. | • Monitoring examinations at regular intervals<br>• Risk of recurrence<br>• Risk that you may still need surgery if the cancer returns<br>• Stress and fear of control examinations<br>• Stool problems (LARS) |

NOT VALIDATED

| | Surgery | Active surveillance |
|---|---|---|
| **Oncological outcome** | | |
| Risk of local recurrence | ≈ 5% | ≈ 30% |
| Chances of being alive after 5 years without metastases or local recurrence | = | |
| Chances of being alive after 5 years | = | |
| Chances that cancer cells have NOT spread to other parts of the body after 2 years | = | |
| **Functional outcome** | | |
| No LARS after 12 months | 26-65%* | 36-67%** |
| Minor LARS after 12 months | 17-50%* | 17-30%** |
| Major LARS after 12 months | 18-56%* | 17-36%** |
| Risk of long-term urinary symptoms (loss of urine, difficulty in completely emptying the bladder, ...) | 30-39% | 22-25% |
| Sexual function | • Women: vaginal dryness, pain during intercourse, decreased libido<br>• Men: erectile dysfunction, ejaculation problems | |

NOT VALIDATED

* Croese et al. Int J Surg. 2018
** Hupkens, et al. Dis Colon Rectum. 2017
** Van der Sande et al. Rad Onc. 2019

## PERSONAL CONSIDERATIONS

Things to reflect on.

| | Totally disagree | | | | Totally agree |
|---|---|---|---|---|---|
| | 1 | 2 | 3 | 4 | 5 |
| I cannot deal with the anxiety and stress that active surveillance may bring. | | | | | |
| Coming for check-ups at regular intervals is difficult to fit into my daily life. | | | | | |
| I think it is important that the affected piece is completely removed. | | | | | |
| I think it is important to avoid the cons of major surgery. | | | | | |
| I find it important to avoid the inconvenience of colostomy care (e.g. pouch emptying, stoma cleaning, etc.). | | | | | |
| I think it is important to avoid the complications of a stoma. | | | | | |
| I cannot cope with the possible impact of a stoma on my daily functioning and self-image. | | | | | |

NOT

| What are you worried about? |
|---|
| |

| What are you worried about concerning the future? |
|---|
| |

| What are your expectations of the treatment? |
|---|
| |

# CONCLUSION

Consider the arguments that are most important to you. Which option do you prefer?

□ Surgery

□ Active surveillance

Your treating physicians can offer support and help you make a choice. You are not alone in this.

Do you have any questions or concerns after reading this decision aid?

You can always contact your treating physicians or nurse specialist. You can also contact other counsellors such as a social worker, psychologist or sexologist.

NOT VALIDATED

## FIND OUT MORE?

Interesting websites:

- https://www.cancer.be/sites/default/files/publication/3-1-13-nl-dikkedarmkanker-02-2017_0.pdf
- https://www.uzleuven.be/nl/rectumkanker

Peer groups:

- https://www.stomavlaanderen.be/
- https://www.stoma-actief.be/
- https://waaiervzw.be/

Ask a healthcare provider about local groups you and your family can talk to.

## QUESTIONS?

If you have any further questions or concerns, you can reach us by phone during office hours at XXX

NOT VALIDATED

© december 2022

**Design and production**

This decision aid was developed as part of a clinical trial.

NOT VALIDATED
